# Supplementary material for: Structural basis for the prolonged photocycle of sensory rhodopsin II revealed by serial synchrotron crystallography
Source: Nat Commun. 2025 Apr 11;16:3460. doi: 10.1038/s41467-025-58263-x (PMC11992208; doi:10.1038/s41467-025-58263-x)
Supplement: Supplementary file 1 — Supplementary Information [file 41467_2025_58263_MOESM1_ESM.pdf]

Structural basis for the prolonged photocycle of Sensory Rhodopsin II revealed by serial synchrotron crystallography

Robert Bosman, Giorgia Ortolani, Swagatha Ghosh, Daniel James, Per Norder, Greger Hammarin, Tinna Björg Úlfarsdóttir, Lucija Ostojić, Tobias Weinert, Florian Dworkowski, Takashi Tomizaki, Jörg Standfuss, Gisela Brändén, Richard Neutze

Supplementary Information

Supplementary information inventory:

Supplementary Figures S1 to S6: pages 2 to 9

Supplementary Tables S1 to S2: pages 2 to 3

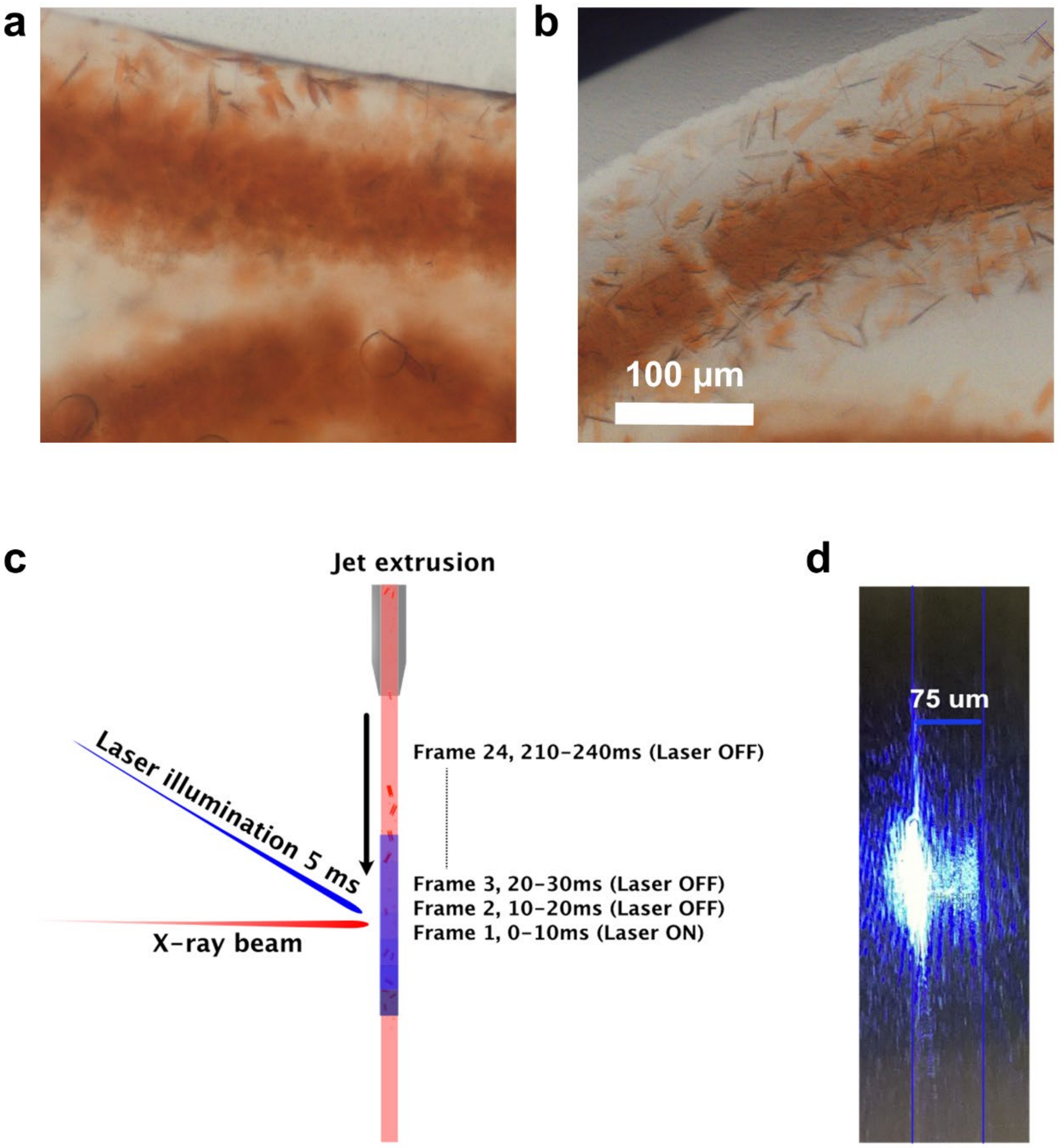

Supplementary Figure 1: Illumination protocol for *Np*SRII microcrystals. **a**) Crystals of *Np*SRII grown in an LCP. **b**) Zoom-in view of these LCP crystals. **c**) Schematic illustrating how microcrystals of *Np*SRII are illuminated by a 5 ms laser burst and then different regions of the microjet that were exposed to this laser pulse move through the X-ray beam as the LCP microjet flows downwards. **b**) Photograph of the illumination of a LCP microjet using a blue laser flash. The boundaries of the LCP microjet are indicated with a blue line.

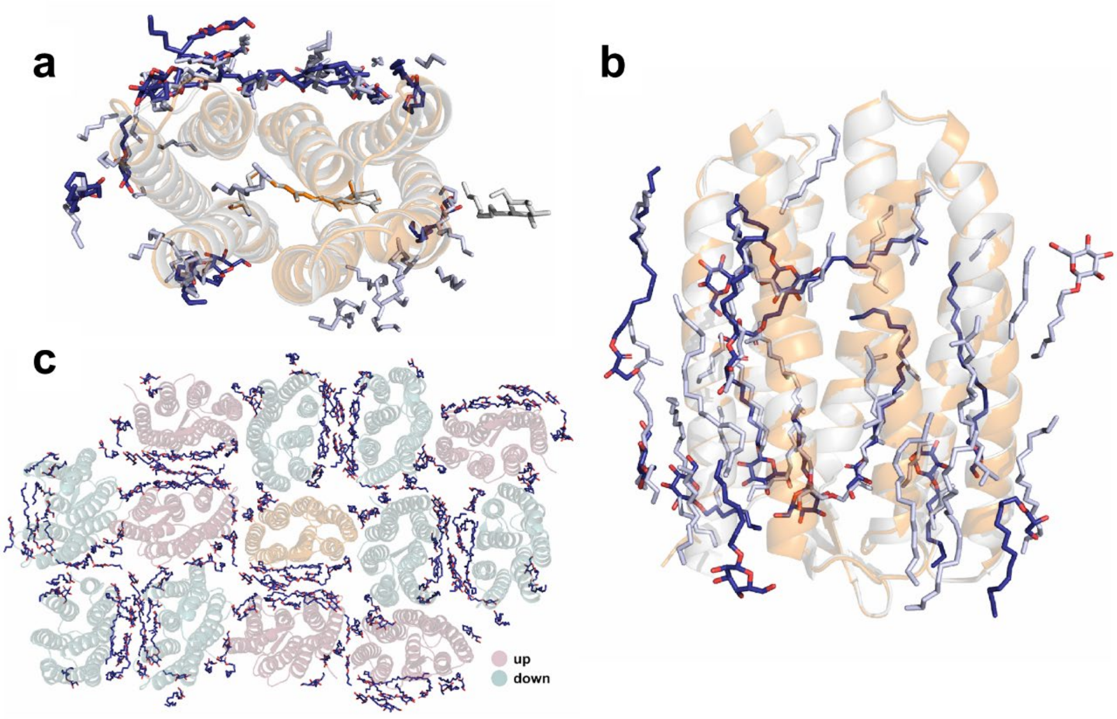

Supplementary Figure 2: Arrangement of lipid molecules in the room-temperature serial crystallography structure of *Np*SRII. **a**) Lipid molecules associated with *Np*SRII (blue) recovered in the room temperature SSX structure (orange) overlaid upon those modelled in an earlier cryo-crystallography structure of *Np*SRII (white) and viewed from above the membrane. **b**) Similar representation as in **a** but viewed from the plane of the membrane. **c**) Crystal packing of *Np*SRII illustrating how lipid molecules are found at the interface between molecules.

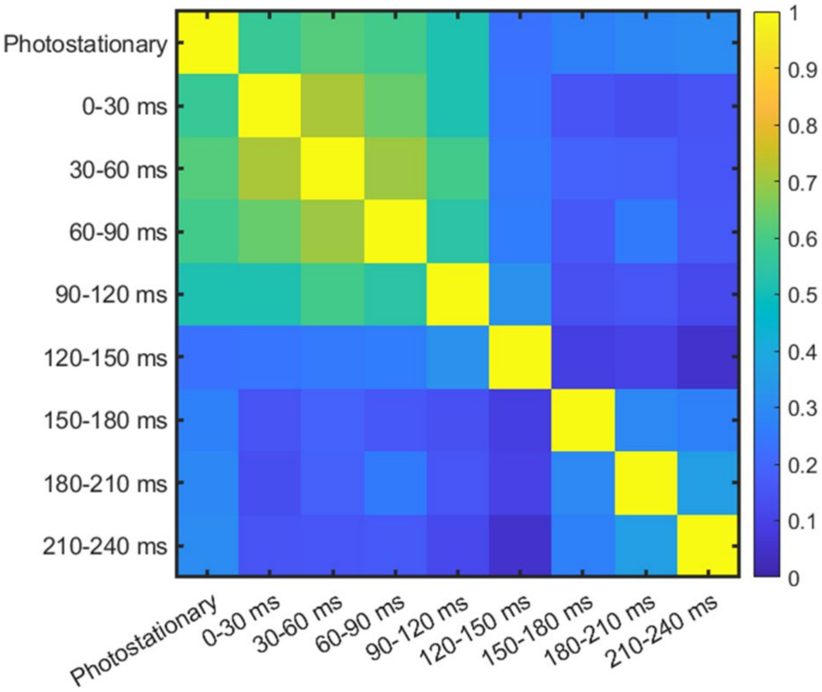

Supplementary Figure 3: Pearson correlation coefficient calculated for the one-dimensional representation of difference electron density shown in **Figure 4a** between the indicated time-delays or during continuous illumination (indicated as Photostationary). Correlations are lost as the movement of the microjet sweeps the light-exposed region out of the X-ray beam. Source data are provided as a Source Data file.

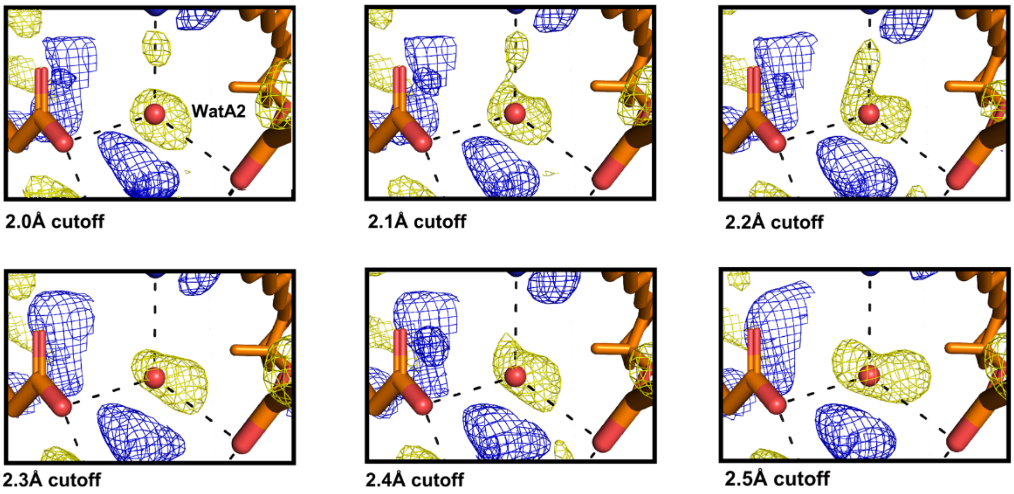

Supplementary Figure 4: The influence of the resolution cut-off (indicated) on the negative difference electron density feature associated with WatA2 of *NpSRII*. These difference Fourier electron density maps are contoured at ±3.0 σ (positive density in blue, negative density in yellow).

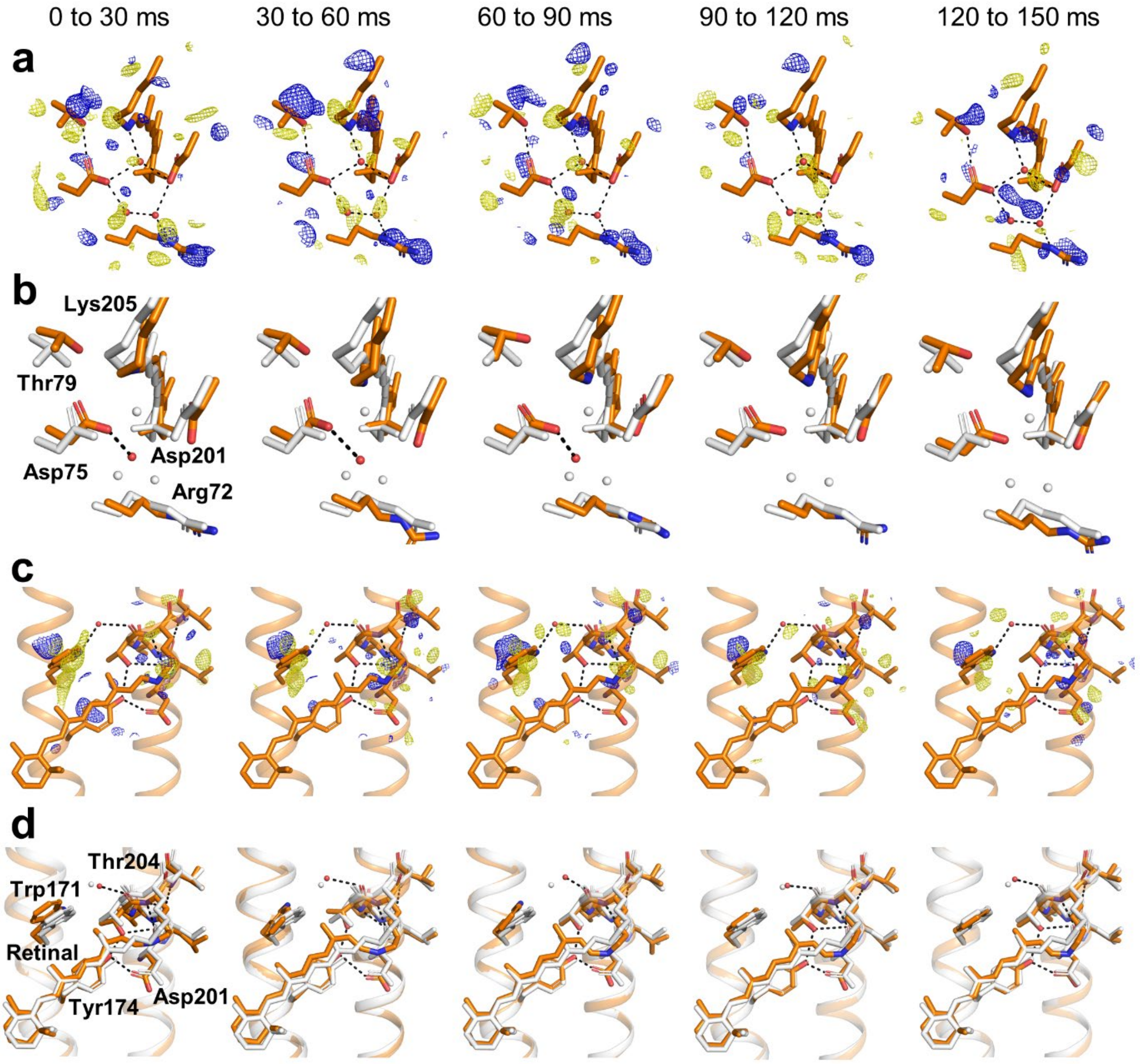

Supplementary Figure 5: Time-dependent electron density and structural changes in *Np*SRII. **a**) F_o(light) − F_o(dark) isomorphous difference Fourier electron density maps (contoured at ±3.0 σ, positive density in blue, negative density in yellow) shown on the extracellular side of the retinal for the time delays Δ*t* = 0 to 30 ms; 30 to 60 ms; 60 to 90 ms; 90 to 120 ms; and 120 to 150 ms. **b**) Refined resting state (white) and photo-excited state (orange) conformations for the time-delays Δ*t* = 0 to 30 ms; 30 to 60 ms; 60 to 90 ms; 90 to 120 ms; and 120 to 150 ms on the extracellular side of the retinal. **c**) F_o(light) − F_o(dark) isomorphous difference Fourier electron density maps (contoured at 3.0 σ) shown on the cytoplasmic side of the retinal for the time

delays Δt = 0 to 30 ms; 30 to 60 ms; 60 to 90 ms; 90 to 120 ms; and 120 to 150 ms. **d**) Refined resting state (white) and photo-excited state (orange) conformations for the time-delays Δt = 0 to 30 ms; 30 to 60 ms; 60 to 90 ms; 90 to 120 ms; and 120 to 150 ms on the cytoplasmic side of the retinal.

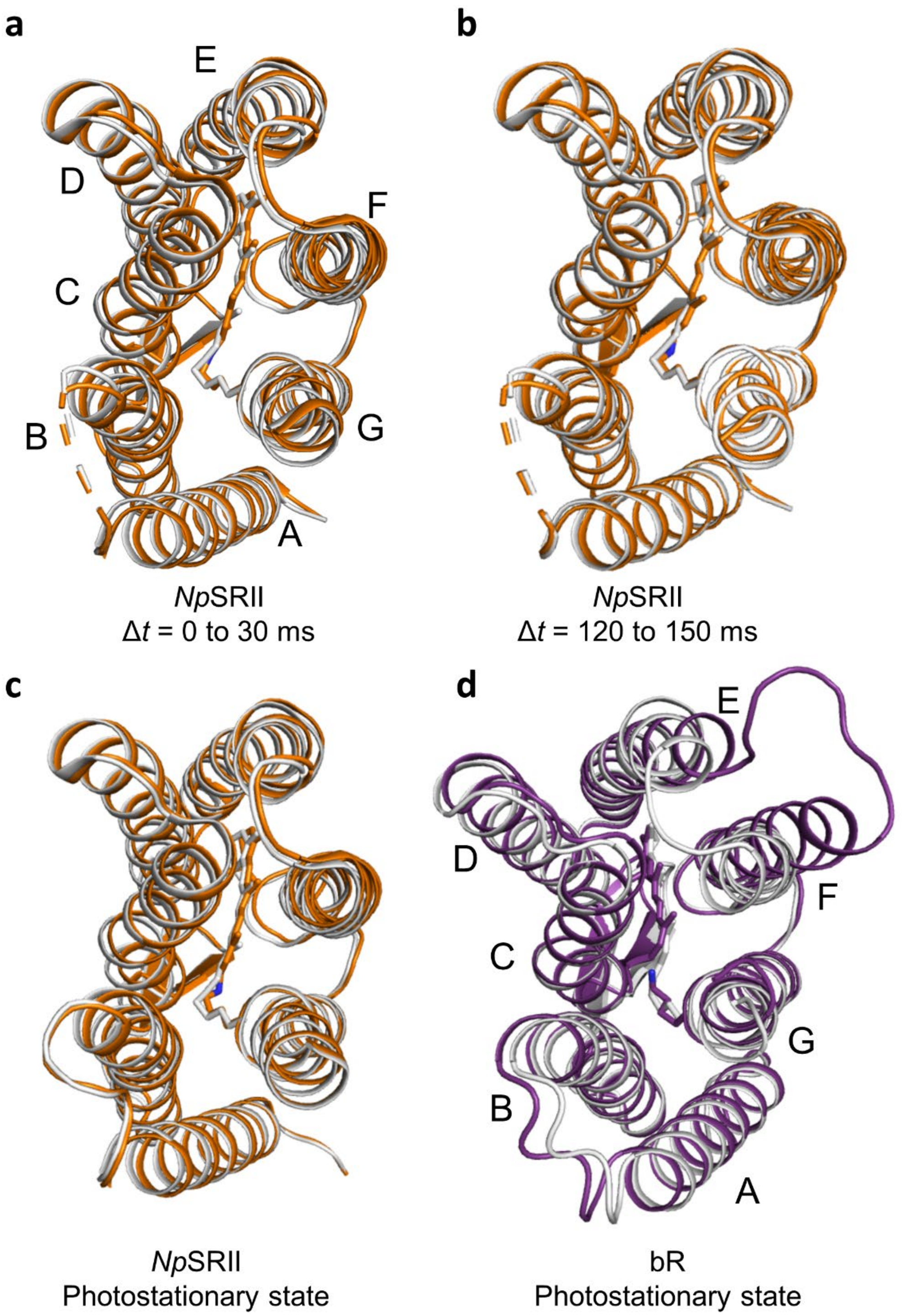

Supplementary Figure 6: Superposition of the resting models and those after structural refinement for *Np*SRII and bR. **a**) Illuminated structure for the time-delay Δt = 0 to 30 ms (orange) superimposed upon the resting conformation (white) of *Np*SRII. **b**) Illuminated structure for the time-delay Δt = 120 to 150 ms (orange) superimposed upon the resting

conformation (white) of *Np*SRII. **c**) Continuously illuminated structure (orange) superimposed upon the resting conformation (white) of *Np*SRII. **d**) Continuously illuminated structure (purple) and the resting conformation (white) of bR.[10]

Supplementary Table 1: X-ray diffraction data collection and structural refinement statistics

	**Dark**	**Illuminated**	**0-30ms**	**30-60ms**	**60-90ms**	**90-120ms**	**120-150ms**	**Grouped dark 150-240**
PDB	9H20	9H1X	8PWP	8PWJ	8PWI	8PWG	8PWQ	9H1W
**Data Collection**								
Collection temperature (K)	293	293	293	293	293	293	293	293
Space group	C2221	C2221	C2221	C2221	C2221	C2221	C2221	C2221
a,b,c (Å)	89.75, 131.7, 51.0	89.75, 131.7, 51.0	89.75, 131.7, 51.0	89.75, 131.7, 51.0	89.75, 131.7, 51.0	89.75, 131.7, 51.0	89.75, 131.7, 51.0	89.75, 131.7, 51.0
α,β,γ (°)	90, 90, 90	90, 90, 90	90, 90, 90	90, 90, 90	90, 90, 90	90, 90, 90	90, 90, 90	90, 90, 90
**Data Statistics**								
Resolution (Å)‡	39.4 – 2.20	44.9 – 1.85	44.9 – 2.4	44.9 – 2.4	44.9 – 2.4	44.9 – 2.4	44.9 – 2.4	44.9 – 2.4
R split (%)†‡	14.0 (147)	15.6 (127)	15.0 (248)	15.0 (197)	14.7 (204)	14.9 (146)	14.5 (111)	8.1 (47.4)
SNR (I/σ(I))‡	5.6 (1.2)	7.3 (0.98)	4.2 (0.39)	4.3 (0.46)	4.5 (0.44)	4.8 (0.62)	5.2 (0.86)	9.3 (1.8)
CC 1/2‡	99.1 (31.0)	99.4 (44.8)	98.7 (16.0)	98.8 (19.0)	98.1 (17.0)	98.1 (28.7)	97.3 (47.0)	99.4 (79.0)
Number of indexed lattices£	26 382	21 518	6 481	6 577	6 890	7 163	7 705	24 485
Number of total reflections	4 385 964	2 879 163	1 620 091	1 605 270	1 722 594	1 811 612	1 965 561	6 360 239
Number of unique reflections	15 729	17 156	12 228	12 419	12 660	13 603	15 036	15 708
Multiplicity‡	279	168	132	129	136	133	131	405
**Refinement**								
Resolution (Å)	39 – 2.2	44 – 2.20	45 – 2.4	45 – 2.4	45 – 2.4	45 – 2.4	45 – 2.4	45 – 2.4
Completeness (%)	99.9 (97)	99.3 (100)	94.6 (79)	96.1 (85)	96.9 (88)	98.9 (96)	99.8 (99)	99.8 (100)
Number of unique reflections	15 729	15 633	11 549	11 732	11 835	12 072	12 177	12 191
R_{work}/R_{free} (%)	20.7/21.8	24.7/26.6	24.9/29.9	24.1/30.6	23.6/27.1	23.7/27.0	22.9/25.6	20.5/20.9
Average B factor (Å^2)	54.0	60.0	45.0	46.0	47.0	45.0	45.0	54.0
Wilson B factor (Å^2)	34.3	35.6	37.0	36.7	37.1	36.3	36.0	47.5
**Crystallographic occupancy€**								
Dark conformation (%)	100	56	63	63	67	79	89	100
Illuminated conformation (%)	0	44	37	37	33	21	11	0
All-*trans* retinal (%)	100	67	64	66	71	82	91	100
13-*cis* retinal (%)	0	33	36	34	29	18	9	0
**R.M.S deviations**								
Bond lengths (Å)	0.008	0.031	0.02	0.017	0.022	0.012	0.012	1.099
Bond angles (°)	1.10	1.48	1.62	1.61	1.79	1.49	1.65	0.01

‡Values for the highest-resolution shells are displayed in parentheses next to the overall value.

†$R_{split} = 1/(\sqrt{2})([\sum hkl|I_{even} - I_{odd}|]/[0.5(\sum hkl|I_{even} + I_{odd}|)])$

£Ratio of the number of indexed images to the total number of images

€The value in brackets represents the crystallographic occupancy of coordinates restrained about the dark structure.

Supplementary Table 2: Population estimates from spectroscopic analysis.

Spectroscopy LCP setups	Activated	Resting	Pop M	Pop O	All-trans
0-30	37%	63%	31%	6%	69%
30-60	35%	65%	22%	14%	78%
60-90	33%	67%	15%	18%	85%
90-120	31%	69%	10%	20%	90%
120-150	28%	72%	7%	21%	93%
Continuous 120 ms	44%	56%	18%	26%	82%
**Spectroscopy Crystals**	**Activated**	**Resting**	**Pop M**	**Pop O**	**All-trans**
0-30	37%	63%	36%	1%	64%
30-60	37%	63%	34%	3%	66%
60-90	37%	63%	33%	4%	67%
90-120	36%	64%	31%	5%	69%
120-150	36%	64%	29%	6%	71%
Continuous 240 ms	44%	56%	33%	11%	67%
**Corrected for translation†**	**Activated**	**Resting**	**Pop M**	**Pop O**	**All-trans**
0-30	37%	63%	36%	1%	64%
30-60	37%	63%	34%	3%	66%
60-90	33%	67%	29%	4%	71%
90-120	21%	79%	18%	3%	82%
120-150	11%	89%	9%	2%	91%
Continuous 240 ms	44%	56%	33%	11%	67%
**Estimate using Xtrapol8**	**Activated**	**Resting**			
0-30	43%	57%			
30-60	32%	68%			
60-90	17%	83%			
90-120	35%	65%			
120-150	13%	87%			
Continuous 240 ms	49%	51%			

†Correction to the population within crystals due to light-exposed sample moving out of the X-ray beam. These values were used to fix the crystallographic occupancy during structural refinement.
