# Supplementary material for: Structural basis for the prolonged photocycle of sensory rhodopsin II revealed by serial synchrotron crystallography
Source: Nat Commun. 2025 Apr 11;16:3460. doi: 10.1038/s41467-025-58263-x (PMC11992208; doi:10.1038/s41467-025-58263-x)
Supplement: Supplementary file 2 — Reporting Summary [file 41467_2025_58263_MOESM2_ESM.pdf]

nature portfolio

Corresponding author(s): Richard Neutze

Last updated by author(s): Feb 27, 2025

Reporting Summary

Nature Portfolio wishes to improve the reproducibility of the work that we publish. This form provides structure for consistency and transparency in reporting. For further information on Nature Portfolio policies, see our Editorial Policies and the Editorial Policy Checklist.

Statistics

For all statistical analyses, confirm that the following items are present in the figure legend, table legend, main text, or Methods section.

n/a	Confirmed	
☐	☒	The exact sample size (*n*) for each experimental group/condition, given as a discrete number and unit of measurement
☒	☐	A statement on whether measurements were taken from distinct samples or whether the same sample was measured repeatedly
☒	☐	The statistical test(s) used AND whether they are one- or two-sided<br>*Only common tests should be described solely by name; describe more complex techniques in the Methods section.*
☒	☐	A description of all covariates tested
☒	☐	A description of any assumptions or corrections, such as tests of normality and adjustment for multiple comparisons
☐	☒	A full description of the statistical parameters including central tendency (e.g. means) or other basic estimates (e.g. regression coefficient) AND variation (e.g. standard deviation) or associated estimates of uncertainty (e.g. confidence intervals)
☒	☐	For null hypothesis testing, the test statistic (e.g. *F*, *t*, *r*) with confidence intervals, effect sizes, degrees of freedom and *P* value noted<br>*Give P values as exact values whenever suitable.*
☒	☐	For Bayesian analysis, information on the choice of priors and Markov chain Monte Carlo settings
☒	☐	For hierarchical and complex designs, identification of the appropriate level for tests and full reporting of outcomes
☐	☒	Estimates of effect sizes (e.g. Cohen's *d*, Pearson's *r*), indicating how they were calculated

Our web collection on statistics for biologists contains articles on many of the points above.

Software and code

Policy information about availability of computer code

Data collection	X-ray diffraction data were collected at the Swiss Light Source (Villigen, Switzerland). This is stated in the acknowledgments.
Data analysis	X-ray diffraction data were analyzed using publicly available code for X-ray diffraction processing and structural analysis. These packages include COOT (version GTK4), Phenix (version 1.21), CCP4 (version 8.0), CrystFEL (version 0.8.0). The programs used and appropriate references/sources are stated in the methods section.<br><br>In-house code written in Matlab (R2024b) used to generate Fig. 2 has been placed on Github at: https://github.com/Neutze-lab/TR_SSX_SRII. Resampling scripts for serial crystallography were described in reference 64 and have been placed at: https://github.com/Neutze-lab/resampling.

For manuscripts utilizing custom algorithms or software that are central to the research but not yet described in published literature, software must be made available to editors and reviewers. We strongly encourage code deposition in a community repository (e.g. GitHub). See the Nature Portfolio guidelines for submitting code & software for further information.

Data

Policy information about availability of data

All manuscripts must include a data availability statement. This statement should provide the following information, where applicable:

- Accession codes, unique identifiers, or web links for publicly available datasets
- A description of any restrictions on data availability
- For clinical datasets or third party data, please ensure that the statement adheres to our policy

Coordinates and structure factors for the SSX NpSRII dark and continuously illuminated structures have been deposited in the RCSB Protein Data Bank under accession codes 9H20 (dark conformation) and 9H1X (continuous illumination), 8PWP (0-30 ms), 8PWJ (30-60 ms), 8PWI (60-90 ms), 8PWG (90-120 ms), 8PWQ (120-150 ms), and 9H1W for the grouped dark time-points (150-240 ms).

Research involving human participants, their data, or biological material

Policy information about studies with human participants or human data. See also policy information about sex, gender (identity/presentation), and sexual orientation and race, ethnicity and racism.

Reporting on sex and gender	Not applicable.
Reporting on race, ethnicity, or other socially relevant groupings	Not applicable.
Population characteristics	Not applicable.
Recruitment	Not applicable.
Ethics oversight	Not applicable.

Note that full information on the approval of the study protocol must also be provided in the manuscript.

Field-specific reporting

Please select the one below that is the best fit for your research. If you are not sure, read the appropriate sections before making your selection.

☒ Life sciences ☐ Behavioural & social sciences ☐ Ecological, evolutionary & environmental sciences

For a reference copy of the document with all sections, see nature.com/documents/nr-reporting-summary-flat.pdf

Life sciences study design

All studies must disclose on these points even when the disclosure is negative.

Sample size	Data were collected at the Swiss Light Source in two separate experiments. Data-set sizes were determined by the beamtime available, the quality of microcrystals, and the volume of microcrystals. We collected as much data as possible (shown in Supplementary Table 1). Since qood quality difference Fourier electron density maps were recovered from these data (Figures 3, 5, 6 and Supplementary Figs. 4 & 5) it was sufficient.
Data exclusions	No data were excluded. Supplementary Table S1 shows that thousands of X-ray diffraction images were successfully indexed and these data were merged for each data-point.
Replication	Two serial x-ray crystallography experiments were performed on two separate occasions at the Swiss light source. Data from continuous illumination were recorded in the first experiment, and data from time-resolved X-ray diffraction were collected at the second experiment. The main structural findings of the work were replicated.
Randomization	All X-ray diffraction data were merged into data-sets summarized in Supplementary Table 1. Thousands of X-ray diffraction images were successfully indexed and these data were merged for each data-point. These were sorted according to no illumination, continuous illumination, or time-resolved data sets.
Blinding	Not applicable. Blinding is not relevant to this study. X-ray diffraction data were recorded at a beamline at the Swiss Light Source from samples produced in the home laboratory and analyzed according to the standards of the field.

Reporting for specific materials, systems and methods

We require information from authors about some types of materials, experimental systems and methods used in many studies. Here, indicate whether each material, system or method listed is relevant to your study. If you are not sure if a list item applies to your research, read the appropriate section before selecting a response.

Materials & experimental systems

n/a	Involved in the study
☒	☐ Antibodies
☒	☐ Eukaryotic cell lines
☒	☐ Palaeontology and archaeology
☒	☐ Animals and other organisms
☒	☐ Clinical data
☒	☐ Dual use research of concern
☒	☐ Plants

Methods

n/a	Involved in the study
☒	☐ ChIP-seq
☒	☐ Flow cytometry
☒	☐ MRI-based neuroimaging

Plants

Seed stocks	Not applicable.
Novel plant genotypes	Not applicable.
Authentication	Not applicable.
